# On the impact of tactile processing on motor cortex: how touch shapes motor behaviour

**DOI:** 10.1007/s00429-026-03128-2

**Published:** 2026-05-25

**Authors:** Antonio Zafarana, Dollyane Muret, Alessandro Farnè, Luigi Tamè

**Affiliations:** 1 School of Psychology, University of Kent, Canterbury, CT2 7NP UK; 2 Integrative Neuroscience and Cognition Center, CNRS UMR 8002, Université Paris Cité, 75006 Paris, France; 3 Impact Team of the Lyon Neuroscience Research Center INSERM U1028 CNRS U5292, University of Lyon 1, Lyon, France; 4 New York University Abu Dhabi, Abu Dhabi, United Arab Emirates

**Keywords:** Somatosensory cortex, Motor cortex, Touch, Tactile training, Sensorimotor integration

## Abstract

The ability to manipulate objects is a fundamental human skill that relies primarily on the motor system. However, effective object manipulation would not be possible without the continuous information provided by the somatosensory system. Cutaneous tactile feedback is particularly important when a movement must be adjusted while performing an action. Efficient interactions between the tactile and motor systems are therefore paramount for fine motor behaviour, as clearly demonstrated by the profound impairments observed following lesions to sensorimotor brain regions. However, somatosensory deficits following cortical damage have received considerably less attention than motor impairments, even though substantial evidence shows that such deficits are typically associated with poorer motor recovery and that preserved somatosensation is a strong predictor of motor outcome. This disconnect highlights a significant gap in the literature: despite the critical role of touch in shaping motor behaviour, the functional relationships between the tactile and motor systems remain inadequately characterized. In this review, we provide a unified (though not exhaustive) synthesis of the evidence pointing towards the substantial role of cutaneous tactile information in modulating motor cortical processing and, consequently, motor behaviour. We first describe, across species, the anatomical and neurophysiological connections linking somatosensory and motor systems and the nature of their interactions. We then review evidence from neuropathological studies demonstrating the severe consequences of disrupted tactile signals on motor performance. Finally, we examine the impact of short- and long-term tactile learning on motor function, highlighting its potential for developing novel tactile-to-motor rehabilitation strategies for individuals suffering from brain injury and other neurological conditions. Table of contents. Introduction 4. Anatomical connections between the somatosensory and motor cortices 8. Functional nature of sensorimotor interactions 13. Neuropathological evidence of sensorimotor interactions 19. Tactile and motor interactions in the context of motor planning and motor learning 26. Effects of tactile training on motor and sensorimotor performance 29. Conclusion and future perspectives 34. References 37.

## Introduction

The ability to manipulate objects plays a crucial role in humans’ everyday life activities. It is suggested that this skill depends on our motor control system communicating with the somatosensory system, which continuously provides information about the position of our body parts, and the status of their skin surfaces (Gentilucci et al. 1997; Scott 2016). Somatosensory and motor systems are indeed extensively inter-connected anatomically (Catani et al. 2012; Balaskas et al. 2020; Baldissera et al. 1981), somatosensory feedback allowing for effective and fast adjustment of movements execution (Moscatelli et al. 2019; Preston and Ehrsson 2016). This is evident in several everyday life situations, whereby precise calculation and balance of forces determined by the interactions between the sensory inputs and motor outputs are used to accurately grasp a mug full of coffee and bring it to the mouth without spilling it out (Johansson and Westling 1984; Mott and Sherrington 1895).

Implementation of action cannot be executed as intended if the somatosensory and motor systems are not communicating effectively (Scott 2016). This has been proven by a series of experiments on monkeys whose afferent sensory signals from both cutaneous and proprioceptive sources were interrupted at different levels of the spinal cord. In a pioneering study, Mott and Sherrington 1895 showed that as a result of hand and foot deafferentation, monkeys were no longer able to perform fine movements (i.e., grasping), whereas deafferentation of the whole forelimb except for the hand and foot made monkeys unable to execute gross movements (i.e., walking) (Mott and Sherrington 1895). From these behavioural observations, the authors concluded that: “afferent impulses, both from the skin and from the muscles, especially the former, as related to the palm and sole, are necessary for carrying out highest-level movements” (Mott and Sherrington 1895, p. 614). Although these early studies did not dissociate tactile from proprioceptive contributions, since then, substantial evidence of interactions between the somatosensory and motor systems has been gathered from a wide range of studies using different approaches and models. Evidence for tactile influences on the cortical component of motor processing and control has often remained distributed across different experimental domains, species, and methodological approaches. Therefore, while most academics in the field acknowledge that touch affects motor cortex activity, this information has not been considered in an integrated and coherent work. Because of the fragmented nature of this information, there has been a limited unified understanding of how touch consistently shapes motor processing, beyond the classical role of proprioception and cutaneous tactile feedback in movement executions.

In this review at first, we wish to clarify and consolidate evidence supporting the importance of the entwined relationship between the cutaneous touch processing and motor processing for human behaviour by analysing some of its key aspects in depth. Note that here we chose to focus primarily on the role of cutaneous touch, setting aside the contribution of other somatosensory processes such as proprioception, temperature perception, affective touch, and pain, which have been extensively discussed elsewhere (Bank et al. 2013; Burns et al. 2016; Chowdhury et al. 2022; Clarke and Harris 2004; Dietz, 2002; Marasco & de Nooji, 2023; Prochazka 2021; Rohel et al., 2021; Yam et al. 2018; Racinais and Oksa 2010; Tamè and Longo 2023; Winter et al. 2022). Nevertheless, due to the methods used in some studies (e.g., median nerve electrical stimulation, which activates nerves containing both cutaneous and muscle afferents), it should be noted that we will also report, some findings involving other somatosensory afferents rather than purely cutaneous inputs. It is important to acknowledge the role of the cerebellum and basal ganglia in sensorimotor processing, as demonstrated by the motor deficits resulting from lesioning these brain structures (Ackerley and Kavounoudias 2015; Diedrichsen and McDougle 2026; Therrien and Bastian 2015; Yin 2017). Moreover, neurons in the spinal cord are essential for both afferent and efferent tactile signals and their integration, however, such topics won’t be the focus of our review as they have been already discussed elsewhere (Groenewegen 2003; Kalambogias and Yoshida 2021; Molinari et al. 2007; Nielsen 2004; Pierrot-Deseilligny 1996; Simonyan 2019). Instead, we will focus specifically on the neocortical interactions involving brain regions such as the primary somatosensory (S1) and motor (M1) cortices and some higher-level regions such as the secondary somatosensory cortex (S2), as less attention has been given to such processing stages.

This manuscript is intended as a narrative review; thus, although not systematically, we relied on PubMed and google scholar searched with the query “(sensorimotor integration* OR somatosensory-motor pathways* OR tactile* OR touch and motor* OR tactile training* OR motor corticospinal excitability* OR somatosensory impairment stroke*) AND (mouse OR mice OR rodent OR monkey OR human)”. Papers were reviewed and then included based on their conceptual relevance to the specific topics discussed in this review. The primary variables assessed were the methods of static tactile stimulation, the evaluation of the motor response, and the reporting of anatomical and physiological information regarding the responding brain areas, for the different species, of the processes. Thus, papers that examined S1-M1 anatomical and neurophysiological connections in mice, monkeys and humans, somatosensory-motor interactions in both healthy and neuropathological populations, and tactile/somatosensory training were reviewed and then included if they met the above content and methodological standard approach. For reasons of conceptual choice and space, although important, in the present review we chose not to include the work involving active touch, such as texture exploration (Lieber and Bensmaia 2022; Simões-Franklin et al. 2011), as well as the role of dynamic skin stretches to generate kinematic illusions (Collins et al. 2005; Landelle et al. 2018 see Proske & Gandeva, 2018 for a review), though, we redirect the reader to the aforementioned references.

To this aim, we will unfold the anatomical and neurophysiological pathways connecting the sensory and motor systems by critically reporting paradigmatic studies in non-human species and neuroimaging works in humans. At the functional level, we will also report and discuss data on the inhibitory and excitatory nature of these connections. In humans, both excitatory and inhibitory pathways between somatosensory and motor cortices have indeed been revealed, for example by work combining cutaneous afferent stimulation and Transcranial Magnetic Stimulation (TMS) (Bikmullina et al. 2009; Kukaswadia et al. 2005; Pilurzi et al. 2013; Sailer et al. 2002; Tokimura et al. 2000). The functional exploration of these sensorimotor connections will then be complemented by insightful cases from neuropathological studies, focusing on patients with stroke (Borich et al. 2015; Frías et al. 2018; Ingemanson et al. 2019; Schaechter et al. 2012; Sullivan & Hedman, 2008) and focal dystonia (Avanzino et al. 2015; Melgari et al. 2013) as paradigmatic examples of the loss of efficient sensorimotor communication. Not last, we will examine the effects of afferent inputs disruption coming from the skin on motor performance. Besides constituting the core of our first aim, these sections will be propaedeutic to achieve the second aim of this review. This consists in providing both empirical and theoretical grounds for developing novel tactile-to-motor rehabilitation strategies, for people suffering from brain injury and other neurological conditions. To this aim, the last section will examine the impact of short- and long-term tactile learning on the motor system. In this section the focus will primarily remain on cutaneous tactile processing, but, because the literature is rather sparse in this field, we will also consider broader somatosensory training paradigms where both cutaneous touch and proprioception are solicited. Most active training procedures typically imply attention-demanding and effortful tasks to be performed intensively for a prolonged period of time. Although these techniques may be effective, they are time-consuming and demanding for people, especially for certain populations such as elderly or stroke patients (Albert and Kesselring 2014; Hunter and Crome 2002; Teasell et al. 2006). Thus, here we will focus on passive learning paradigms and particularly on repetitive somatosensory stimulation (RSS), which applied at the tip of one or multiple fingers has been proven to be effective in modulating tactile performance. Likely relying on the interactions that will be detailed in the first sections of this review, the potential effects produced by RSS training on sensorimotor performance, and the more limited direct motor benefits, will be discussed. Altogether, this review will provide a much-awaited state-of-the-art of the key functional aspects of sensorimotor crosstalk and their neural underpinnings and will hopefully pave the way to the development of much-needed innovative, sensory-to-motor therapeutic approaches for clinical populations.

### Anatomical connections between the somatosensory and motor cortices

The somatosensory and motor systems are anatomically densely interconnected both within and across hemispheres, a feature consistently documented in several species. Indeed, several studies on non-human species (Aronoff et al. 2010; Kinnischtzke et al. 2016; Krubitzer and Kaas 1990; Mao et al. 2011) and human primates (Berlot et al. 2019; Catani et al. 2012; Eickhoff et al. 2010), have disclosed the underlying neuroanatomical connections between sensory and motor brain regions.

In this section, we adopt a phylogenetic approach, discussing first results from species that are evolutionarily distant from humans (i.e., mice), before focusing on macaques, a popular non-human primate model for studying the neural basis of human dexterity, finishing with reports on humans. Although this approach does not cover all lineages of the phylogenetic tree (Krubitzer and Seelke 2012), it is motivated by the close similarity between macaque and human prehension abilities (see Yan et al. 2021 for a review).

While mice, monkeys, and humans share comparable patterns of intra- and inter-hemispheric connectivity, they differ greatly in terms of their anatomical organization and brain areas differentiation. The mouse cortex is dominated by large primary and secondary somatosensory and motor areas, with the former having round-like arrangements. In contrast, monkeys exhibit a greater degree of cortical parcellation, and their somatosensory cortex is organized as a strip-like sequence of areas (3a, 3b, 1, 2), similar to that observed in humans (Van Essen et al., 2019). Despite these anatomical differences, recent quantitative comparative approaches have enabled more precise cross-species alignment, facilitating the translation of findings from non-human models to humans (Beauchamp et al. 2022; Mars et al. 2018, 2021).

Evidence of dense connections between the sensory and motor systems was provided by a study that examined the long-range connections of the mouse’s primary somatosensory barrel cortex, which is the cortical region receiving signals from the whiskers through the thalamus (Aronoff et al. 2010). Other studies showed that S1 (postcentral gyrus) axons project to M1 (Petrof et al. 2015) and vice versa, demonstrating the existence of reciprocal connections between these two brain areas (Kinnischtzke et al. 2016; Mao et al. 2011; Petreanu et al. 2009; Rocco-Donovan et al. 2011; Ferezou et al. 2007). Complementing these findings, anatomical studies using injected tracers reported direct neuronal projections from S1 to M1 in rodents (Bedwell et al. 2014) and primates (Krubitzer and Kaas 1990; Stepniewska et al. 1993). In particular, specific areas of the primary somatosensory cortex (e.g., area 1) has been shown to have direct projections to the primary motor cortex (Pons and Kaas 1986; Rosen and Halgren 2021). Motor performance may be critically dependent on somatosensory cortex: in rats, it drives the activation of motor cortex through intracortical projections (Farkas et al. 1999), and in mice, it is engaged in dynamic cortical interactions to flexibly integrate sensory input with motor output (Ferezou et al. 2007). In line with this perspective, Karadimas and colleagues (2020) showed that S1 pyramidal neurons’ activity, which preceded movement onset, was highly correlated with locomotor speed in mice. In a similar vein, Chang and colleagues (2022) showed that silencing the inputs that S1 receives from S2 significantly disrupt the hind paw movements during locomotion in mice (Chang et al. 2022). Indeed, in addition to the reciprocal S1 and S2 connections, there are sensorimotor circuits involving M1 and S2 (Smith and Alloway 2013; Suter and Shepherd 2015), as well as interhemispheric connections between the two S2 (Carvell and Simons 1987). These findings show that S1 activity in mice can affect motor output, using a direct S1-to-effector pathway or through other motor-related areas.

**Fig. 1:**
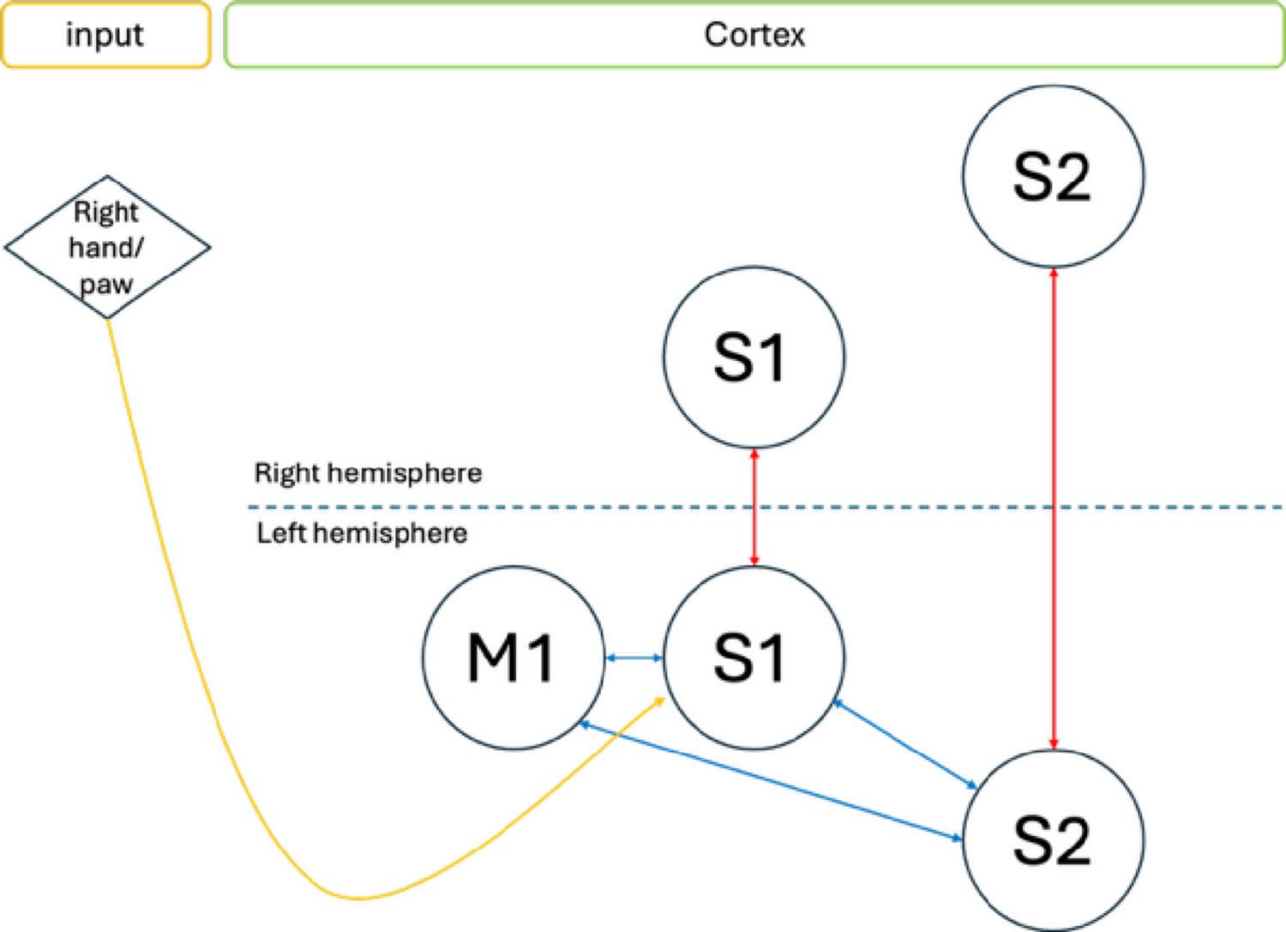
Schematic representation of the connections between S1, M1 and S2 shared across mice, macaques and human cortices. In mice, intra-hemispheric connections between S1 and M1 appear to be crucial for motor behaviour with S2 providing additional information for correct movement execution (see in text). In monkeys, somatosensory signals from either S1 or peripheral afferent neurons play a significant role in both movement initiation and modulation. Finally, both intra-hemispheric and interhemispheric connections were found in humans using DTI and post-mortem examinations. Blue arrows correspond to intra-hemispheric connections whereas red arrows correspond to the interhemispheric ones

While rodent models have been instrumental in identifying basic principles of sensorimotor organization, non-human primates and in particular macaques, provide a closer translational model both for neocortical organization and manual dexterity. Extensive anatomical connections between somatosensory and motor cortices have also been documented in this species. Tract tracing studies unveiled an intricate pattern of intra- and inter-hemispheric connections between S1 and M1 (Burton et al. 1995; Disbrow et al. 2000; Gharbawie et al. 2011; Ghosh et al. 1987; Jones et al. 1978; Krubitzer et al. 1986; Krubitzer and Kaas 1990; Künzle 1978; Morecraft et al. 2015; Pons and Kaas 1986; Stepniewska et al. 1993) and their respective projections to S2 (Krubitzer and Kaas 1990; Künzle 1978), showing a similar pattern of connectivity as in mice (Fig. 1). S1 is indirectly connected to M1 in the contralateral hemisphere also via the transcallosal connections between the two S1 and via S2 which is highly connected to S1 and M1 (Krubitzer and Kaas 1990). Furthermore, S2 has reciprocal ipsilateral connections with S1 and M1 as well as with the contralateral S2 (Krubitzer and Kaas 1990) (Fig. 1). At the functional level, intracortical microstimulation (ICMS) of S1 has been found to evoke movements in primates (Baldwin et al., 2018) and to affect recorded muscle activity (Widener and Cheney 1997), often of inhibitory nature. Reciprocally, recordings of M1 neural activity in awake monkeys demonstrated that a group of neurons was clearly responding to afferent tactile stimuli on the fingers or hand when there were no movements (Lemon 1981). Authors also suggested that some M1 neurons may receive cutaneous inputs directly from the thalamus without relying on S1, as demonstrated by the short latency of the response (around 10 ms). This indicates that cutaneous afferent inputs may directly affect the motor cortex activity (Rosén and Asanuma 1972). Further evidence was provided by Yazdan and colleagues (2018), who showed that stimulating a small region in S1 or M1 using optogenetics, strengthened the connectivity (measured by stimulus-evoked response ratio and coherence) between these two areas (Yazdan-Shahmorad et al. 2018). Finally, several studies examined the laminar and topographic connections between the somatosensory and the motor cortices revealing more details regarding the specific projections between areas 1, 2, 3a, 3b and area 4 (M1) (Burton and Fabri 1995; Darian-Smith et al. 1993; Jones et al. 1978; Pons and Kaas 1986) as well as the spatial organization of neural activity related to movements such as grasping and reaching within these areas (Friedman et al. 2020). Such connections are important for supporting precise sensorimotor maps that allow faster adjustment and real-time feedback for performing skilled movements.

While the human literature is less extensive and methodologically different, several human studies (Entz et al. 2014; Osborn et al. 2021; Rosen and Halgren 2021) have provided indirect evidence consistent with the finding of intra-hemispheric S1-M1 connections found in non-human species. For instance, Catani and colleagues (2012) investigated the structural intra-hemispheric connections of the pre- and post-central gyri using diffusion tensor imaging (DTI) in conjunction with a post-mortem examination. This method revealed the existence of several groups of u-shaped tracts connecting S1 and M1 homuncular representations, such as for instance the paracentral lobule tract connecting S1 and M1 “foot” representations and the superior, ventral and transverse tracts connecting S1 and M1 at the level of the hand area (Catani et al. 2012). Eickhoff et al. 2010 analysed the intra-hemispheric anatomical and functional connectivity of S2 using both probabilistic tractography on diffusor tension imaging (DTI) data and a meta-analytic connectivity modelling on a functional database. They found the presence of tracts linking S2 and S1, as well as between S2 and M1. The functional analysis revealed significant coactivation (i.e., connectivity) of S2 (seed region) with S1, M1 and the premotor cortex. Regarding the inter-hemispheric structural connectivity, which is thought to support inter-hemispheric inhibition/excitation processing (Ni et al. 2009), the examination of the transcallosal tracts connecting homologous S1 and M1 regions using DTI (Fling et al. 2013) revealed moderate S1 inter-hemispheric connection, and dense fibre tracts connecting M1 regions (Fig. 1). There are also interhemispheric connections between the S2 regions (Chung et al. 2014; Stancak et al. 2002). Note that diffusion-based tractography cannot confirm direct monosynaptic projections in the same way as tracer injection methods used in non-human animal studies; therefore, findings in humans should be interpreted with caution regarding the potential existence of direct connections. In addition to the homotopic (same brain areas) interhemispheric connections discussed so far, recent evidence in humans and other species has demonstrated the existence of heterotopic (different brain areas) interhemispheric connections linking non-homologous cortical areas of the two hemispheres (Szczupak et al. 2023), within S1 and between S1 and M1. Taken together, the studies described in this section provide compelling and converging evidence that the somatosensory and motor systems have strong interconnections with one another both at the intra- and inter-hemispheric levels.

### Functional nature of sensorimotor interactions

In this section, we examine the functional properties of the connections described above and the mechanisms through which sensory and motor systems exchange information. Specifically, we consider the excitatory and inhibitory nature of these pathways, the factors that modulate them and how they interact with each other (Table 1). Ultimately, we synthesize the empirical evidence suggesting the functional role served by these dense interconnections.

It has been shown that if electrocutaneous stimulation of peripheral nerves close to a target muscle precedes a TMS pulse over M1, the resulting motor evoked potential (MEP) from the targeted muscle may be inhibited or facilitated (Di Lazzaro et al. 2005; Tokimura et al. 2000; Udupa et al. 2009). For these reasons, TMS is considered an ideal tool to assess sensorimotor functions (Reis et al. 2008; Staines and Bolton 2013; Turco et al. 2018). The following section reports on studies whereby TMS has been applied over the M1 hand area while MEPs were recorded from the first dorsal interosseous (FDI), unless specified otherwise. The emergence of excitatory and inhibitory effects is modulated by factors such as stimulation onset, type of afferent nerves (mixed or cutaneous), somatotopic distribution (homotopic or heterotopic stimulation), stimulation intensities (both peripheral and TMS) and specific neurotransmitters involved (GABA, ACh) (Turco et al. 2018). Each of these factors, which provides evidence on the mechanisms underlying the interactions between S1 and M1, will be discussed in detail in this section.

Before discussing these factors, it is important to note a methodological caveat. Several studies of afferent modulation of M1 involve median nerve stimulation, which recruits a mixed afferent volley and therefore does not isolate purely cutaneous inputs. These studies are informative about somatosensory-motor interaction but should not be taken as evidence specific to cutaneous touch, as can be done for cases using cutaneous digital nerve stimulation.

The importance of stimulation onset has been demonstrated by Delwaide & Oliver (1990), who showed that peripheral stimulation reduced MEP amplitude evoked by single-pulse TMS applied on the contralateral M1 when the interstimulus interval (ISI) between the sensory afferent stimulus and TMS is short (about 20ms) or long (about 200ms). The former is called short-latency afferent inhibition (hereafter SAI), and the latter long-latency afferent inhibition (hereafter LAI) (Chen et al. 1999). By using TMS while recording corticospinal volleys in patients with implanted electrodes in the cervical epidural space, Tokimura and colleagues (2000) found that the afferent input inhibits motor corticospinal excitability as early as 5ms from the arrival of the input to the cortex, suggesting rapid cortical sensorimotor interactions (possibly thalamo-cortical pathway) (Tokimura et al. 2000).

The type of afferent nerves involved in SAI has been explored by Bayle and colleagues (2016) using either median nerve or cutaneous digital nerve stimulation to examine the relationship between the type of afferent sensory fibres (mixed or purely sensory), somatosensory evoked potentials (SEP) and SAI. They found that SAI is positively correlated both with the recruitment of sensory afferent fibres and somatosensory excitability, as measured by variations in SEPs amplitude (Bailey et al. 2016). This suggests that the magnitude of MEP inhibition (i.e., SAI) depends on both the sensory afferent volley (i.e., fibres recruited) and S1 excitation (i.e., SEPs). It is important to note that the factors described here are not exhaustive, and they can also interact with each other. For example, median nerve stimulation (a type of afferent stimulation) can inhibit MEPs if delivered at specific onsets (around 20 ms), but it can also facilitate MEPs at other onsets (between 45 ms and 70 ms) (Devanne et al. 2009).

**Table 1:**
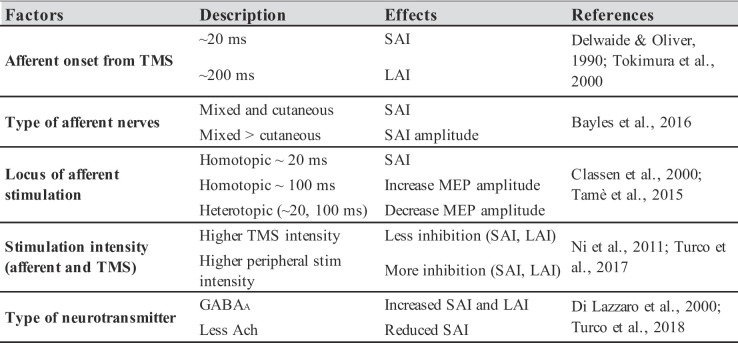
List of the described empirical evidence that play a role in the interactions between the somatosensory and motor cortices

The third factor/parameter affecting SAI is the somatotopic distribution. The stimulation is considered homotopic if the afferent stimulation is applied near the targeted muscle of the TMS (e.g., MEP recorded from FDI and afferent stimulation of index finger or thumb) and heterotopic if it is far from the targeted muscle (e.g., MEP recorded from FDI and afferent stimulation on the little finger). To analyse how somatotopic distribution affects sensorimotor integration, Classen and colleagues (2000) applied electrical stimulation on the right thumb or little finger of one hand while delivering a TMS pulse over M1. They measured the abductor pollicis brevis (APB) and abductor digiti minimi (ADM) muscles MEP responses at different inter-stimulus intervals between the afferent stimulus and the TMS pulse (Classen et al. 2000). Results showed that *homotopic* conditioning stimulation caused inhibition of the MEP at short ISI (20ms) and excitation of the MEP at long ISI (100ms), whereas *heterotopic* stimulation caused inhibition at both ISIs although the inhibition was weaker for the short interval (Classen et al. 2000). These results suggest that this interaction is mediated by the state of the muscle (somatotopic gradient for relaxed but not contracted) and that cutaneous and motor signals are integrated in a somatotopically organised fashion, though, the timing between the afferent stimuli can alter such organization (Tamè et al. 2015). A neuroimaging (functional magnetic resonance imaging, fMRI) study in humans, using a repetition suppression paradigm (Tamè et al. 2012), showed a finger-specific response in S1 following the contralateral tactile stimulation of the fingers (index and middle fingers). Intriguingly, the authors also found a deactivation pattern in M1 that was mirror symmetric to the finger-specific response in S1. Following up on this study, Tamè and colleagues combined a similar adaptation paradigm with a SAI protocol in which they applied TMS over M1 to investigate how spatial and temporal aspects of tactile inputs may affect motor outputs/excitability (Tamè et al. 2015). They found that electrocutaneous stimulation of the same finger lessened the amount of MEPs inhibition compared to different fingers, specifically at short ISIs. On the other hand, MEPs were significantly more inhibited with a long ISI between the tactile stimuli both when stimulation occurred on the same or different pairs of fingers (i.e., index-index vs. middle-index). These results suggest that the time between stimuli is a crucial factor in determining how the somatosensory information impacts M1 (or more in general cortico-spinal excitability) processing, the short and long intervals resulting in different patterns of inhibition. These patterns of transfer can be important for informing/tuning fine motor skills (Johansson & Flanagan, 2009).

A fourth factor influencing the excitatory and inhibitory sensorimotor interaction is stimulation intensity. Increasing TMS intensity has been shown to modulate the afferent inhibition effect (Ni et al. 2011). This is probably because a higher TMS intensity stimulates a greater number of neurons thus the inhibition induced by somatosensory afferent inputs struggles to exert the same effect over a larger population activity. At the same time, the intensity of the median nerve stimulation has also been demonstrated to significantly regulate the mixed (cutaneous and proprioceptive) afferent inhibition strength (Ni et al. 2011; Turco et al. 2017). These and related findings were observed thanks to the implementation of protocols such as the short-interval intra-cortical inhibition (SICI) and the long-interval intra-cortical inhibition (LICI), which used TMS to assess inhibition in the human motor cortex. In these paradigms, a subthreshold TMS pulse, called conditioning stimulus (CS), is applied shortly (1-5ms), or a supra-threshold TMS pulse later (50-200ms), before a supra-threshold TMS pulse called test stimulus (TS), both delivered to M1. Conversely, if the ISI between the CS and the TS is within 8-15ms, intra-cortical facilitation (ICF) is observed (Kujirai et al. 1993; Ziemann et al. 1996). Moreover, short-latency intracortical facilitation (SICF) is elicited when the CS and TS are peri- or supra-threshold and applied with an ISI of 1-3ms (Tokimura et al. 1996).

The neuromodulation processes involved in SAI have been suggested to be related to GABA activity, which appears to modulate cortical excitability through inhibition of cholinergic transmission (Metherate and Ashe 1995). Some results support the idea that inhibition involving GABA modulates the task-related activity of motor neurons (Matsumura et al. 1992). Based on this information, Di Lazzaro and colleagues (2000) tested whether inhibitory and excitatory circuits between the somatosensory and motor systems are GABA-dependent or not. The authors found that scopolamine, which is an anticholinergic agent, significantly reduced the resting motor threshold, as well as the effects of the SAI produced by median nerve stimulation. However, the administration of scopolamine did not seem to affect either SICI or SICF, suggesting that these mechanisms rely on different neuromodulators (Di Lazzaro et al. 2000). Since then, several studies corroborated these results suggesting that SAI is likely to rely on a cholinergic pathway that is modulated by GABA (Müller-Dahlhaus et al. 2008; Turco et al. 2018). Altogether, these studies provide important information that can help delineate the neuronal pathway that is used to integrate somatosensory (cutaneous and proprioceptive) and motor signals by knowing the receptors that affect their patterns of excitation and inhibition (SAI, SICI, LICI).

**Fig. 2:**
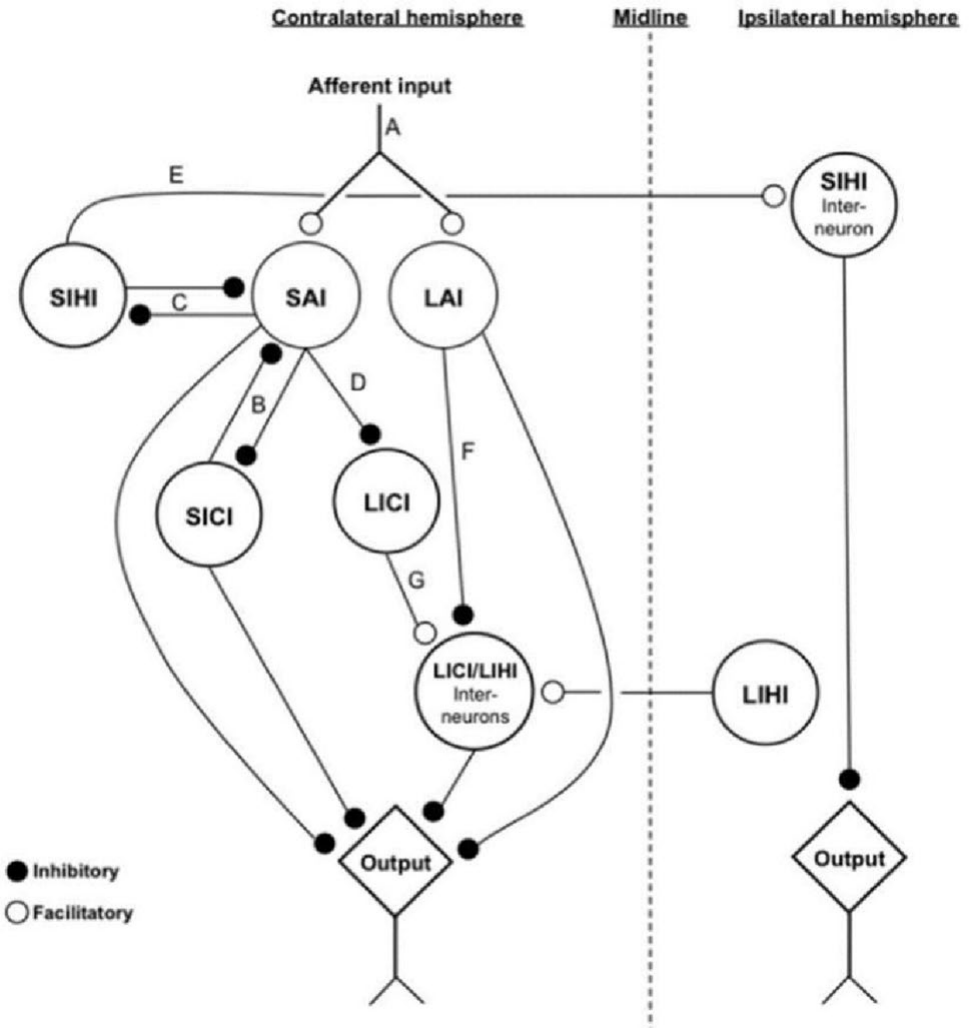
Working model of somatosensory inputs and motor outputs interactions [Taken from Turco et al. 2018**]**. Working Model of the interactions between the somatosensory inputs and motor outputs analysed by using the TMS. The dotted line defines the midline between the left and right hemispheres. The black circles indicate inhibitory connections while the white circles represent excitatory connections. In this example, afferent inputs (**A**) arrive at the contralateral hemisphere (i.e., right hand = left hemisphere) activating SAI or LAI (based on ISI) which then inhibit the motor output while interacting with other motor processes such as SICI (**B**) and LICI (**D**) in the case of SAI, or a population of inhibitory connections (**F**) in the case of LAI

Finally, the inhibitory and excitatory interactions between sensory and motor stimuli deeply influence each other, as demonstrated by several studies (Alle et al. 2009; Kukaswadia et al. 2005; Sailer et al. 2002; Udupa et al. 2009). These results suggest that somatosensory inputs have an impact on motor processing although it is not clear whether these afferent inputs influence M1 directly (thalamocortical) or via S1 (corticocortical) (Turco et al. 2018) for a review).

In conclusion, somatosensory afferent inputs significantly reduce motor outputs when delivered at specific timings (SAI, LAI). Moreover, the impact of M1 inhibition can be somatotopically or not somatotopically distributed depending on S1 excitability and on the features (i.e., timing, location and intensity of the afferent stimuli) of the sensory afferent signals. Furthermore, thanks to the intracortical inhibitions in M1 and their interactions with the afferent inhibitions (SAI, LAI) we were able to comprehend that these processes underlie, at least in part, different structures/pathways (see also Fig. 2). Finally, the studies we described demonstrate that the mechanisms underlying facilitation and inhibition interactions between S1 and M1 are based on cholinergic and GABAergic neurons’ activity.

### Neuropathological evidence of sensorimotor interactions

**Fig. 3:**
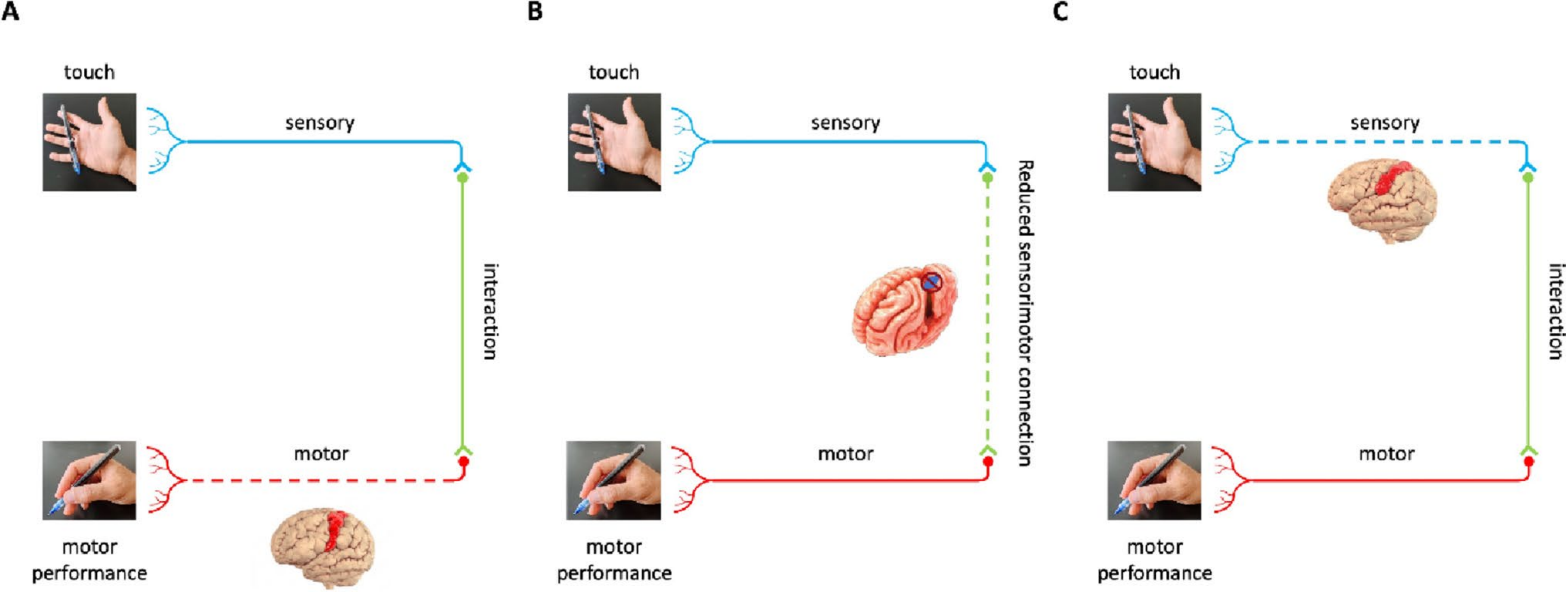
This illustration offers a concise overview of a few potential causes of motor performance impairments. Dotted lines represent the lesion site. The left image (**A**) illustrates that a lesion to the primary motor cortex can be a cause. The middle image (**B**) shows that reduced connectivity between the somatosensory and motor systems may affect motor performance. Finally, the right image (**C**) indicates that lesions to the primary somatosensory cortex can also lead to a motor performance decrease

Since S1 and M1 are highly interconnected and influence each other (see the section on anatomical connections), it is expected that neurological conditions or brain lesions affecting either region would alter sensorimotor performance (Fig. 3). How important are the peripheral tactile inputs arising from the skin? In this section we will start by examining how disrupting somatosensory signals at the periphery can affect motor performance. Notably, the evidence presented in this following section does not uniquely point towards direct (S1-M1 projections) or indirect pathways (S1-Thalamo-M1, or S1-subcortical-M1 circuits) for the motor output modulation, but rather a combination of them.

A substantial body of evidence shows that removing tactile inputs, through peripheral anaesthesia or deafferentation, significantly disrupts motor performance, in particular fine motor coordination (Augurelle et al. 2003; Gentilucci et al. 1997; Johansson and Westling 1984; Monzée et al. 2003; Moscatelli et al. 2019). Seminal work using two closely related precision-grip paradigms demonstrated that anesthetising the thumb and index finger - leaving mostly motor and proprioceptive signals unaffected - impaired the ability to appropriately scale grip and load forces to different surface frictional properties of objects (Johansson and Westling 1984), and increased object slippage by 30% relative to the control (no anaesthesia) condition (Gentilucci et al. 1997).

Cardinali et al. (2016), by testing a patient with right upper limb deafferentation, showed that there was a severe alteration in the kinematic profile of the patient’s deafferented (absence of proprioceptive and tactile inputs) arm when reaching objects (i.e., multiple peaks of acceleration and deceleration). Deafferented patients with large-fibre sensory loss (both tactile and proprioceptive) in the upper limb were found to display altered kinematics when grasping and lifting various objects, with slower movements and larger grip apertures compared to healthy individuals (Miall et al. 2019). Moreover, patients often adopted postures that eased visual guidance and simplified control complexity. Similarly, TMS applied over the left motor hand area showed that anaesthetic block of the right median and radial nerves at the level of the wrist reduced MEP amplitudes in the FDI muscle (deprived of cutaneous signals), while leaving MEPs in the ADM muscle (whose cutaneous signals remained intact) unchanged (Rossi et al. 1998). The authors interpreted these results as proof of the impact of tonic tactile inputs coming from the skin surrounding the contracted muscle on the corticospinal excitability of that specific muscle (since their absence decreased its MEPs). This modulation was probably mediated by thalamo-cortical projections to S1 and subsequent S1–M1 interactions, with direct thalamo–M1 inputs likely playing a secondary or modulatory role, as suggested by more recent evidence (Turco et al. 2018; Rosen and Halgren 2021).

Deafferentation can also be the result of limb amputation, in which both sensory inputs to S1 and muscle targeted from M1 are not available. This lack of signals has long been associated with maladaptive plasticity (e.g., Flor et al. 1998; Raffin 2021). However, the last decade of research in the field highlighted the existence of distributed representational content (or latent activity) throughout the Homunculus (e.g., face representational content being detectable in the foot region; Muret et al., 2022) and that deprivation, by uncovering such pre-existing latent activity (Wesselink et al., 2022), can manifest as remapping, though probably fostering functional stability (i.e. homeostasis) rather than reorganisation of the system (see Muret and Makin 2021; Makin and Krakauer 2023). Notably, the vast majority of amputees experience - at least for some time - phantom sensations (Stankevicius et al. 2021). While their neural substrate is still not fully elucidated, converging evidence points out the persistence of the S1 hand representation in amputees (Mercier et al. 2006; Kikkert et al. 2016; Wesselink et al. 2019). Interestingly, enhancing tactile inputs in amputees using transcutaneous electrical nerve stimulation to elicit phantom sensations matching hand grips was found to induce faster information transmission from S1 to M1 (Ding et al. 2020). Similarly, restoring tactile inputs in upper-limb amputees (Valle et al. 2018) or in tetraplegic patients (due to spinal cord injury; Flesher et al. 2021; Serino et al. 2022) using either peripheral or intracortical (S1) stimulation was found to improve prosthesis dexterity, force discrimination, and object manipulation (Valle et al. 2018), robotic arm control (Flesher et al. 2021), and led to higher sense of agency, confidence of control, and improved BMI efficiency (action control) (Serino et al. 2022).

While fascinating, we will not discuss this aspect of tactile restoration further as the development of sensory feedback in prosthetics as a promising avenue for the full integration of a missing body part has been already elegantly discussed elsewhere (Ackerley and Kavounoudias 2015).

Next, we will discuss how brain lesions can affect the interaction between the somatosensory and motor system. Leveraging very selective experimental lesions in M1 of mice, Fukui and colleagues (2020) showed that S1 processing was impaired during the acute phase of the infarct (corresponding to less than 30 days after stroke in humans). Thanks to the combination of anatomical tracing, electrophysiological recordings, computational modelling, and optogenetic manipulation, the disruption of corticortical inputs from M1 (especially inhibitory projections) and the relative damage of layer specific sensory processing in S1, strongly supports a causal functional coupling between the two cortices.

In humans, stroke is the most common (and studied) neuropathological model of brain lesions. The sensory dysfunction experienced by people after stroke has been reported to be one of the important prognostic factors for the recovery of their motor functions (Carey 2017; Kessner et al. 2016; Reding and Potes 1988). This is supported by the fact that somatosensory-evoked potentials (SEPs) in stroke patients can be predictive of motor recovery (Kusoffsky et al. 1982; Coupar et al. 2012). Somatosensory evoked fields (SEFs) recorded using MEG from S2 contralateral to the affected hand of patients who suffered from an ischemic stroke that damaged either the parietal lobe (spared S1 and S2) and insula, or subcortical areas, were correlated with the results of two motor tests (Pegboard Test and Action Research Arm Test (ARAT) measuring hand motor function (such as grip, grasp) 1–7 days, 3–4 weeks and 3 months after stroke (Forss et al. 2012). Despite its limitations in terms of damaged regions (relatively large) and sample size (relatively small), the results from this study support the notion that S2 may be involved in modulating the tactile afferent input to M1 used to accomplish a successful sensorimotor integration process. Converging neuroimaging evidence further underscores the critical role of tactile processing and of S1-related tracts integrity for an effective post-stroke sensorimotor recovery. Using fMRI, Schaechter and colleagues (2012) showed that tactile stimulation of the affected hand evoked greater BOLD responses in the ipsilesional sensorimotor cortex compared to controls, and that this activity was positively correlated with motor recovery of the affected arm (Fugl-Meyer Stroke Scale) between 3 and 4 weeks and 3 months after stroke. Complementing these functional findings, DTI in individuals with chronic stroke and heterogeneous lesion volume and location revealed significant reductions in the microstructural integrity of ipsilesional thalamus-S1, thalamus-M1, and interhemispheric S1-S1 pathways (Borstad et al. 2016; Fig. 3B). Importantly, the integrity of these tracts strongly predicted performance of the affected hand in a haptic perceptual task (HASTe), accounting for up to 90% of the variance in the HASTe scores. While somatosensory tracts’ integrity alone explained most of the observed variance, as expected from a task relying heavily on tactile perception, these findings highlight the tight structural and functional coupling between somatosensory and motor systems that is needed for haptic performance and motor recovery after stroke. While the integrity of S1-M1 tracts was not assessed in this study (likely due to the extent of some lesions), it is important to note that direct S1-M1 connections (see the section on anatomical connections) could also play a role in such effects.

Abnormalities in somatosensory and motor integration have also been suggested to play an important role in other disorders such as focal dystonia, which is characterized by involuntary movements either at rest or during action execution (Fahn et al. 1998). Focal hand and cervical dystonia are two common forms of dystonia and several studies have explored the mechanisms and neural basis underlying them (Albanese et al. 2013; Avanzino et al. 2015). Tactile spatial acuity is reduced in patients with focal hand dystonia as measured by the two most common spatial discrimination tasks: the two-point discrimination task (2PDT), in which two probes separated by different distances are applied to the skin of the participant who has to tell whether they have been touched by 1 or 2 probes (though see Craig & Johnson, 2000); and the grating orientation task (GOT), in which the orientation of tactile/texture gratings of varying width has to be discriminated (Molloy et al. 2003; Sanger et al. 2001; Van Boven and Johnson 1994). Tasks requiring somatosensory and motor integration such as reaching a specific object with the upper limb without vision suggested that patients with focal hand dystonia have an impaired integration of proprioceptive information with motor kinematics (i.e., longer deceleration phase and more inaccuracies) (Inzelberg et al. 1995). These behavioural and perceptual abnormalities are consistent with impaired sensorimotor integration mechanisms, where altered processing/weighting of somatosensory inputs (i.e., temporal/spatial discrimination deficits) results in an impaired calibration of the motor command (Abbruzzese and Berardelli 2003). This sensory processing misalignment contributes to maladaptive plasticity in sensorimotor circuits, resulting in a ‘smearing’ of representational zones in the somatosensory cortex that causes a decrease of motor inhibition (Bara-Jimenez et al. 1998; Byl et al. 1996; Elbert et al. 1998). Therefore, when considering sensory discrimination and proprioceptive processing deficits, they should not simply be perceived as related symptoms but as significant contributing factors to a broader sensorimotor communications dysfunction associated with dystonic movement patterns’ onset and persistence (Quartarone & Hallett., 2013). Since then, several studies confirmed that sensorimotor integration, proprioceptive information, and temporal/spatial somatosensory discrimination all play important roles in focal dystonia (Conte et al. 2019; Desrochers et al. 2019; Konczak and Abbruzzese 2013).

Other interesting phenomena that can be observed after brain lesions include misoplegia, which consist of a strong aversive behaviour towards a limb; asomatognosia, which is characterized by the lack of awareness of a body part; and somatoparaphrenia, a condition in which the ownership of the affected body part is not acknowledged, but instead attributed to someone else (Loetscher et al. 2006; Vallar and Ronchi 2009). The latter phenomenon has been explained as an issue in updating the dynamic representation of the body (body schema), which leads to the sense of disownership of the contralesional limb when its spatial location has been changed and inversely, a sense of ownership for a limb positioned in the still non-updated spatial location (Garbarini et al. 2017, 2020; Romano and Maravita 2019). The erroneous representation might also derive from a disconnection between the primary information of position sense (proprioception and vision) and the areas processing the remapping of that. Romano and Maravita (2019) suggested that such condition may derive from a disconnection between the primary sensorimotor regions and associative brain areas.

The somatosensory system thus appears to actively interact with the motor cortex, but the details regarding the information exchange are still not clear. Umeda and his team (2019) examined the signals S1 receives during reaching and grasping from both afferent neurons and M1 in awake monkeys, using simultaneous electrodes arrays and EMG. Their results showed that before movement initiation, S1 activity was decoded from M1 whereas during movements S1 activity was accounted for both M1 and afferent activities. S1 also encodes information regarding the future limb state. This information then interacts with upcoming sensory feedback signals, thus helping the processing of somatosensory-motor integration in real-time (Umeda et al. 2019).

The evidence provided by the neuropsychological studies we reported, highlights the critical role that the somatosensory system and sensorimotor integration play in motor functions. Therefore, structural and functional brain changes, as well as inhibitory and excitatory interactions between somatosensory and motor areas, are essential aspects to consider when examining any motor outcome. Moreover, this evidence provides indications that the somatosensory system plays a critical role in the recovery of motor functions.

These findings collectively indicate that there is evidence of adaptive tactile-motor plasticity as a result of long-term expertise and short-term learning. In summary, the evidence presented in the latter section demonstrates that tactile input arising from the periphery is also essential for motor ability.

### Tactile and motor interactions in the context of motor planning and motor learning

Tactile and motor interactions are also observed under adaptive conditions such as motor planning and motor learning (e.g., Gale et al. 2021; Vidoni et al. 2010). Indeed, in this section we will discuss a series of empirical evidence suggesting the influence of touch on motor control, specifically planning and learning of an action.

When planning a voluntary movement, for instance grasping a water bottle, the forward model predicts the future somatosensory input (e.g. mechanoreceptors activation), using the efference copy and the current hand/body state (i.e. joint angles, muscle stretch, limb position). The effects of S1 processing and touch on motor control can be clearly seen from a recent study by Serino and colleagues (2022). Using intracortical brain-machine interface (BMI), they showed that M1 activity encodes information regarding intended movement before movement execution, whereas the activity after the movement implementation encodes the sensory feedback associated with that action.

While M1 activity is known to encode tactile information after movement onset (Serino et al. 2022), it is striking to note that the effector-related information could be decoded in the contralateral areas 3b as well as bilateral 1 and 2 already during motor planning (Gale et al. 2021). This suggests that during voluntary movements, the motor cortex prepares and shares an efference copy with S1, which might prepare it for the somatosensory information that will be received during the movement.

According to the comparator model, after movement execution, the somatosensory feedback is combined with the prediction to estimate the new hand state, and generate a sense of agency (Carruthers, 2012). In the case of self-induced touch, the predicted afferent signal leads to perceiving touch as less intense than when induced by others (Blakemore et al. 1999; Brooks and Cullen 2019), and this sensory attenuation has also been used as an implicit measure of the sense of agency. This correlation is further supported by research on schizophrenia, where patients exhibit reduced sensory attenuation compared to healthy controls (Shergill et al. 2005, 2014).

Complementary evidence of adaptive plasticity in tactile-motor integration can be seen in healthy individuals both after acquiring a skill over time (Gärtner et al. 2013; Elbert et al. 1995) and following a short duration of practice (Rosenkranz and Rothwell 2006; Stefan et al. 2000). Musicians are a prime example of how experience can change sensorimotor coupling. Compared to non-musicians, musicians have an increased size of representation of the fingers in the somatosensory area of the brain and greater ability for tactile discrimination than non-musicians, indicating how long-term reweighting of tactile input supports skilled use of their hands (Elbert et al. 1995; Ragert et al. 2004). Musical practice also leads to structural differences compared to matched controls, for instance gray matter in S1 and M1 (in addition to other areas) has been seen to scale with practice intensity (Gärtner et al. 2013; Gaser and Schlaug 2003). In addition, musicians show altered SAI reflecting functional changes of tactile-motor coupling (Hirano et al. 2020). Evidence from neuroimaging studies demonstrates that practice changes the way musicians’ brains engage the sensorimotor networks, suggesting altered interactions between their sensory and motor systems (Bangert et al. 2006; Herholz and Zatorre 2012). At shorter time scales, findings from experimental studies reveal that sensory and motor interactions can change quickly while acquiring new sensorimotor skills. Brief periods of sensorimotor training modulate the balance of excitatory and inhibitory influences within the somatosensory and motor areas, modify corticospinal excitability, and demonstrate dynamic adjustments to S1–M1 integration (Rosenkranz and Rothwell 2006; Stefan et al. 2000; Ridding and Rothwell 1999). These findings collectively indicate that there is evidence of adaptive tactile-motor plasticity as a result of long-term expertise and short-term learning.

Peripheral somatosensory inputs have also been suggested to be important factors for motor learning (Matur and Öge 2017; Van Breda et al. 2017). The current framework of motor skill learning, distinguishes between two mechanisms of motor skill acquisition: the use-dependent mechanism, which occurs when the performance of a task is reinforced through the process of repeated activation of the individual’s sensory and motor pathways and/or muscles; and the error-based mechanism, which relies on the generation of errors while completing a physical movement and the resulting prediction error as feedback (Diedrichsen et al. 2010). In this respect, it has been shown that the sensory component in these two mechanisms may play a significant role in shaping the effectiveness of motor learning. Indeed, Ohashi et al. 2019; found that motor learning is associated with an enhanced S1 excitability (SEPs), which correlates with the gain in the behavioural motor performance (Ohashi et al. 2019). Interestingly, at the initial stages of the learning process, authors did not find any correlation between the behavioural motor performance and other measures of motor excitability such as MEPs. This suggests that it is unlikely that additional mechanisms, such as attentional modulation and/or plasticity-related motor changes, played a key role in the effect at this early stage. However, they do suggest that later stages of the learning process involved a more prominent role of motor excitability, as they found significant changes in the MEPs responses.

TMS has been used to temporarily interfere with the excitability of a cortical area in healthy people by delivering repetitive pulses following specific protocols, allowing to study the functional role of specific brain regions, without inducing any lesion. One of these protocols is repetitive TMS (rTMS) that consists of trains of TMS pulses interleaved with a pause and can be delivered at different frequencies and intensities. By applying either, a sham or a 1 Hz rTMS over the S1 in healthy individuals during implicit motor learning, Vidoni and colleagues (2010) were able to show that interfering with S1 processing significantly altered motor performance (Fig. 3C). This suggests that S1 is necessary for the formation of an accurate motor plan/internal model of movements sequence (Vidoni et al. 2010). Further investigation on motor learning by Kumar et al. (2019) revealed that delivering continuous theta burst TMS (cTBS, 3 pulses at 50 Hz every 200ms for 40 s) on S1 immediately after an implicit motor learning task almost eliminated retention, but did not alter retention when applied after 24 h, once learning was already consolidated. Moreover, cTBS over M1 following learning had little effect on retention thus indicating that S1 plays a crucial role in implicit motor learning consolidation. Recent work further supported the essential role of S1 in motor learning. For instance, Ebrahimi and colleagues (2024) found that disruption of S1 activity using cTBS impaired motor memory retention, whereas cTBS on M1 had no impact. Therefore, authors suggest that S1 is involved in the retention and encoding of newly learned movements following visuomotor adaptation. Similarly, Wang et al. (2024) used cTBS to disrupt activity in S1 or M1 right after participants performed an implicit sequence learning (execute motor actions in a specific order) and dynamic adaptation (adapting motor commands to novel conditions) tasks. They found that consolidation of sequence learning was dependent on M1 but not on S1, whereas the consolidation of dynamic adaptation was reliant on S1 but not on M1.

Together these findings suggest a functional dissociation between S1 and M1, where the former stabilises and encodes sensory-related motor updates and the latter consolidates sequential motor actions.

### Effects of tactile training on motor and sensorimotor performance

A particularly interesting phenomenon that we believe deserves attention in the context of the role of the somatosensory system on motor function is the effect of tactile learning. In the previous sections, we have presented evidence of the somatosensory and motor systems connections and their excitatory and inhibitory interactions. Moreover, neuroimaging, neuropathological and behavioural studies discussed so far showed that tactile processing can affect motor behaviour thanks to S1-M1 intra- and inter-hemispheric connectivity through inhibition, excitation, and haptic information. Based on this knowledge, tactile learning becomes important to this review because it can effectively alter somatosensory functions and this may have, in turn, a significant impact on motor function.

The effects of tactile training have been investigated in patients with focal hand dystonia (Zeuner et al. 2002), since this disorder seems to result from impairments in both somatosensory and motor functions. The training consisted of Braille reading for 1 h/day for 5 days the first week and then 30 min/day for 7 weeks. Motor functioning was assessed using both the Fahn dystonia scale, which indicates the arm dystonia disability, and a standard paragraph writing. After training, patients had increased spatial tactile acuity (almost comparable to healthy controls) and improved motor performance.

Tactile stimulation (trunk and foot) can be used to improve dynamic balance performance (postural control) among older individuals, with the ability to discriminate tactile vibratory stimulation correlating with balance stability (Tanaka et al. 2025). Using haptic perception as a training produces moderate-to-large improvements in manual dexterity in patients with developmental coordination disorder (Wuang et al. 2022). In addition, it was found that tactile stimulation during motor imagery increased neural representation and sensory processing in the motor cortex and improved decoding (performance) in BCI applications (Zhong et al., 2022).

An interesting perspective is provided by a passive tactile stimulation procedure, called repetitive somatosensory stimulation (RSS). Such approach allows precise experimental control over the duration, intensity, and spatial targeting of cutaneous input and, most importantly, is effective without having participants attending to the stimulation or performing a task (Godde et al. 1996, 2000). RSS is a 20 min to 3 h long protocol that is thought to rely on Hebbian synaptic plasticity mechanisms. While not consistent across studies, tactile improvements were in some cases positively correlated with cortical changes in the contralateral S1 with respect to the locus of stimulation (Dinse et al. 2003b; Godde et al. 2003; Höffken et al. 2007; Pleger et al. 2003). Interestingly, RSS has been reported to improve tactile perception not only at the stimulated sites, but also at physically distant, unstimulated body-parts such as the face and the other hand (Muret et al. 2014, 2024).

**Fig. 4:**
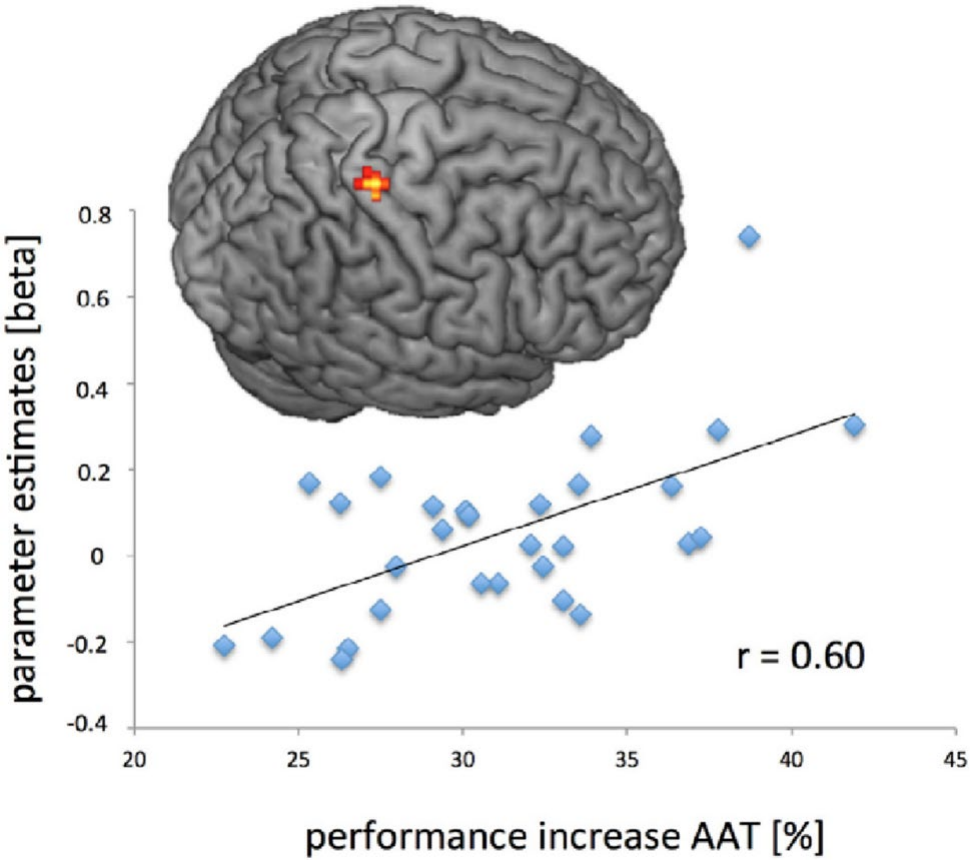
Illustration of the linear regression analysis between the fMRI activation in the contralateral S1 after the RSS stimulation and the percentage of sensorimotor performance improvement measured by the Arm Ability Training (AAT). The figure is reproduced from Lotze et al. (2017) with kind permission from *Brain Stimulation*

Other studies have explored possible RSS effects on sensorimotor performance. For instance, Kalisch and colleagues (2008) applied RSS on all fingers of the right hand in neurologically typical elderly participants, whose somatosensory and motor abilities were impaired compared to young adults. Results showed improved performance in a fine haptic exploration task (without vision) as well as in two pegboard tasks (with short and standard pins) after RSS. When RSS was protracted for 2 days a week over the course of 4 weeks, the gains in both fine sensorimotor (pegboa rd) and haptic performance were maintained respectively for up to 1 and 2 weeks after RSS (Kalisch et al. 2010). However, the tactile domain showed the most lasting improvements, with participants’ spatial acuity (2PDT) returning to baseline level after 1 week. Converging evidence comes from a study that assessed in young adults the added value of RSS applied on the non-dominant hand when combined (or not) with a comprehensive 1 h long daily motor training called Arm Ability Training (AAT) repeated over two weeks (Lotze et al. 2017). Results showed that the stimulation significantly increased contralateral S1 activity and participants’ performance gain in all AAT tasks (see also Fig. 4). These results suggest that RSS may be used together with other sensorimotor training techniques to further enhance gain in arm ability performance in healthy individuals, although the extent to which such gain would reflect a direct motor improvement, or improved tactile sensitivity/sensorimotor processing, remains unsolved.

However, at this stage it is unclear whether RSS might be beneficial for sensorimotor recovery in stroke patients. Research using RSS in clinical and translational studies among populations with neurological disorders has identified both the promise as well as the challenges associated with tactile-based interventions. Evidence from early pilot studies conducted with brain injured individuals was used to demonstrate the potential benefits of RSS on specific aspects of motor performance, focusing on the effector involved in the task (Smith et al. 2009; Kattenstroth et al. 2018). Nonetheless, many of these studies have demonstrated limitations associated with small sample sizes (e.g., Pleger et al. 2001; Hodzic et al., 2004; Kalisch et al. 2008; Smith et al. 2009), and variability between individuals in terms of outcomes (Godde et al. 2000; Dinse et al. 2003a; Höffken et al. 2007; Kattenstroth et al. 2018).

In addition to the limitations associated with existing research, there are three major methodological issues that constrain the interpretation and generalizability of results related to RSS. First, most studies characterized improvements based on outcome measures that involve both sensory and motor components, making it challenging to isolate direct benefits on motor function alone. Second, not all RSS studies have used a systematic comparison to an active sham intervention, making it difficult to make strong causal inferences regarding the relationship between RSS and improved performance. Finally, the long-term effects of RSS appear to be highly dependent on the stimulation protocol (e.g., site, duration, strength) (see Lesemann et al. 2015). Overall, these issues indicate that while RSS was consistently found to enhance tactile processing, the potential of RSS to enhance motor performance in individuals remains to be investigated, as well as the path to translating these effects into reliable and long-term benefits for motor performance.

Nevertheless, a major advantage of RSS is that it does not require attention or active participation to effectively drive plasticity. In addition, the previously reported remote effects of RSS on tactile acuity have a promising rehabilitative potential, making it possible to tap into preserved parts of the sensorimotor system, targeting unaltered body parts (i.e., face or intact hand), to promote recovery in the affected limb. Indeed, Muret and colleagues (2014) have shown the presence of RSS-induced perceptual changes across body-parts that, though physically remote, are cortically close to the stimulated site, such as the hand and the face (Muret et al. 2014). These findings suggest that RSS-induced plasticity might modulate a broader set of brain areas than initially thought, without necessarily being dependent on, or limited by, cortical proximity (Muret et al. 2016; Muret and Dinse 2018). Therefore, it can be particularly suitable in cases in which patients have restricted mobility and/or are not capable of performing active training.

Taken together, the evidence indicates that tactile-based training paradigms, including RSS, have been shown to induce plasticity in the somatosensory system and to modulate sensorimotor interactions. However, many behavioural outcomes are sensorimotor rather than purely motor; therefore, the evidence does not allow any firm conclusion regarding the motor origin of these improvements. In addition, the magnitude and consistency of motor effects are unpredictable based on the current data, suggesting that motor specific outcome measures, adequate control conditions, and long-term follow up will be necessary for future studies to determine exactly when and how tactile-based interventions can meaningfully impact motor function and to define their potential utility within sensorimotor rehabilitation models.

## Conclusion and future perspectives

In this review, we delineated the multilevel impact that the somatosensory system has on the motor system. We have detailed the S1 and M1 reciprocal connections and how S1 activity can directly affect motor performance and M1 activity. Moreover, we have highlighted a consistent pattern of both intra- and interhemispheric connections between S1, M1 and S2 across multiple species (mice, monkeys and humans) from several studies that used a wide range of techniques. Furthermore, the studies presented have shown that somatosensory afferent stimuli can significantly modulate motor excitability if delivered at a specific time, and that S1 excitability affects motor output. Finally, these processes were shown to be related to neurotransmitter activity GABA and ACh proving important clues to understanding the neural pathways subserving these somatosensory and motor interactions. From the neuropathological cases reported we also observed the importance the somatosensory system plays in sensorimotor integration. Indeed, somatosensory activity and the integrity of the somatosensory brain areas and thalamocortical tracts in patients with brain lesions or neurological disorders has become a well-established, important factor for their motor recovery.

The significance of sensorimotor integration for the accurate movement’s execution has been consistently confirmed by studies exploring different aspects of somatosensory information, such as touch or proprioception. Combining the knowledge regarding the relationship between the somatosensory and motor systems with synaptic plasticity, tactile stimulation studies have tried to induce cortical and behavioural changes. From the evidence presented, RSS has shown that passive tactile training can significantly modulate the somatosensory system both at the cortical and perceptual levels. Although the effect of repetitive passive mechanical stimulation on motor activity remains to be explored in full, we believe that it is a very promising field of study for opening novel therapeutics (e.g., support motor abilities’ recovery, stimulate S1 when M1 is damaged, and stimulate other body-parts remotely), and thus deserves further investigation.

RSS should be further evaluated to establish whether it can become a useful technique in the context of sensorimotor recovery in elderly or stroke patients since touch has been proven to be essential for fine manual dexterity. In the future, the effects of RSS on both motor corticospinal excitability and motor performance (measured by a purely motor task instead of sensorimotor ones) should be further explored to unfold the mechanisms of the somatosensory and motor systems communication.

Finally, a continuing issue involves evaluating the temporal dynamics and directionality of communication between S1 and M1 during motor behaviour. Several studies have shown that tactile inputs affect motor cortex excitability; however, only a few have examined how sensory and motor signals interact during motor learning or movement execution. To clarify this aspect, scientists need to use electrophysiological evidence that can combine peripheral stimulation with direct cortical recordings or causal interventions like TMS or intracranial stimulation. This method can separate fast thalamo-cortical effects from the S1-M1 cortical pathways and reveal the roles of inhibitory and excitatory circuits. This would lead to a better understanding of the mechanistic relationship between behavioural changes (i.e., performance) and the neural pathways involved in achieving a specific motor task.

In addition to summarizing and unifying previous evidence on tactile effects, this review identifies a broader conceptual framework: that tactile processing has a more central role in motor cortical function rather than only being an additional source of feedback that is used by the motor cortex to improve the quality of movement execution. Across anatomical, neurophysiological, neuropathological and training-related evidence, tactile signals consistently affect motor cortical excitability, timing and integration in the distributed sensorimotor cortical circuits. Specificity, temporal dependence and context-sensitivity of these effects may explain the variability in the tactile effects on motor behaviour reported in different studies. Such variability is unlikely to result from weak or unreliable tactile-motor connections but rather likely to result from their highly complex interplay by nature; the impact of touch on motor processing being dependent on the stimulation parameters used, task requirements, learning stage, and functional states of the networks. Recognising this conditional structure would allow to unify the interpretation of previous studies and establish a systematic basis for future fundamental and translational research on using tactile mechanisms to facilitate motor activity. Overall, cutaneous inputs do not merely provide corrective feedback but actively shape motor cortical processing, positioning touch as a fundamental component in motor behaviour.

## Data Availability

Since no datasets were generated or analysed during this review, data sharing is not applicable to this article.
